# Assessment of comorbidities, risk factors, and post tuberculosis lung disease in National Tuberculosis Guidelines: A scoping review

**DOI:** 10.1371/journal.pgph.0004935

**Published:** 2025-07-23

**Authors:** Muhammed S. Bah, Kyaw Ko Ko Htet, Gregory P. Bisson, Celso Khosa, Refiloe Masekela, Jamilah Meghji, Kagiso Mochankana, Andrea Rachow, Neelima Navuluri

**Affiliations:** 1 Department of Population Health Science, Duke University, Durham, North Carolina, United States of America; 2 Department of Epidemiology, Faculty of Medicine, Prince of Songkla University, Hat Yai, Songkhla, Thailand; 3 Department of Medicine, Perelman School of Medicine at the University of Pennsylvania, Philadelphia, Pennsylvania, United States of America; 4 Instituto Nacional de Saúde, Marracuene, Mozambique; 5 Department of Clinical Sciences, Liverpool School of Tropical Medicine, Liverpool, New York, United Kingdom; 6 Department of Paediatrics and Child Health, School of Clinical Medicine, College of Health Sciences, University of KwaZulu-Natal, Durban, South Africa; 7 Africa Health Research Institute, Durban, South Africa; 8 National Heart and Lung Institute, Imperial College London, London, United Kingdom; 9 Division of Infectious Diseases and Tropical Medicine, University Hospital, LMU Munich, Munich, Germany; 10 German Center for Infection Research (DZIF), Partner Site Munich, Munich, Germany; 11 Unit Global Health, Helmholtz Zentrum München, German Research Center for Environmental Health (HMGU), Neuherberg, Germany; 12 Division of Pulmonary, Allergy and Critical Care, Department of Medicine, Duke University, Durham, North Carolina, United States of America; 13 Duke Global Health Institute, Duke University, Durham, North Carolina, United States of America; Imperial College London / North Bristol NHS Trust / University of Heidelberg, UNITED KINGDOM OF GREAT BRITAIN AND NORTHERN IRELAND

## Abstract

Tuberculosis (TB) remains a major public health issue across the world and national TB guidelines are an important resource for diagnosis and treatment. This scoping review aimed to analyze how countries with the highest TB burdens approach the integration of comorbidity and risk factor screening, diagnosis and treatment, TB recurrence, and post-TB lung disease (PTLD) diagnosis and management, within their TB guidelines. We used the Arksey and O’Malley methodological framework to conduct a scoping review of TB guidelines among the WHO list of highest-TB burden countries. We identified drug-susceptible, drug-resistant, and consolidated guidelines through web searches and personal contacts within TB programs. We translated guidelines into English as needed and systematically extracted, recorded, and reviewed the guidelines to aggregate and describe our findings. Among the 49 countries with the highest TB burden, we successfully identified, translated, and analyzed 43 guidelines (24 drug-sensitive, 9 drug-resistance, and 10 consolidated) from 34 countries. Recommendations for screening varied by comorbidity or risk factor with the four most recommended being HIV/AIDS (100%), pregnancy (73%) and liver disease (59%) and mental health (59%). Recommendations for linkage to care were more infrequent and also varied with the top four being HIV (88%), liver disease (47%), diabetes (44%), and mental health (44%). Only 27 (79%) countries specified diagnostic tests to assess for TB recurrence among individuals presenting with symptoms post-TB treatment, with 25 recommending GeneXpert MTB/RIF. Notably, only 7 (21%) countries mentioned PTLD in their guidelines, with wide variations in their specific recommendations regarding screening, diagnosis, and management. Our findings highlight the lack of detailed guidance on how to properly diagnose and refer patients to appropriate care for various comorbidities or risk factors which may significantly impact microbiological and clinical TB treatment outcomes, including PTLD and ultimately point to an important opportunity for improvement in future guidelines.

## Introduction

Tuberculosis (TB) is a significant public health concern affecting over 10 million people annually [1]. In 2023, most TB cases occurred in the World Health Organization (WHO) regions of South-East Asia (45%), Africa (24%), and the Western Pacific (17%) [2]. The WHO’s global TB program aims to “lead and guide the global effort to end the TB epidemic,” and provides key recommendations and strategies regarding TB prevention, diagnosis and treatment [3], helping standardize and disseminate recommendations to clinical providers to help achieve the End TB Strategy [4] goals of reducing TB burden and death [5].

National TB control programs (NTPs) rely on country-specific guidelines in addition to WHO guidelines. Country-level guidelines are typically developed via a thorough, multi-step process in which a committee comprising of TB experts reviews existing scientific literature, international guidelines, and national and regional TB epidemiological data to inform recommendations [6]. Country-level guidelines often differ from those of the WHO since countries prioritize specific facets of TB care depending on their local health policies and goals, epidemiological and health infrastructure, and availability of tests and medications [7]. Once finalized, guidelines are disseminated and integrated into practice, and countries carry out monitoring and evaluation to gauge adherence to the guidelines and assess their impact on TB management. Country-level guidelines can therefore significantly impact a country’s progress towards End TB Strategy goals and gaps or variation between different countries’ guidelines may help inform future public health and policy efforts.

WHO and NTP TB guidelines have multiple evolving areas. For example, the WHO recently published a consolidated guideline for TB-HIV [8], given the increased risk of TB among people living with HIV, [9] and the negative impact of TB disease on HIV progression, [10,11]. WHO also released guidelines dedicated to TB comorbidities, which as of 2025, addresses mental health, HIV, and diabetes [12]. These comorbidities, along with risk factors such as tobacco and alcohol use, can have substantial impacts on the risk of developing TB, unsuccessful treatment outcomes (e.g., death and treatment failure), and long-term lung function [13–22]. Yet, the inclusion of comorbidity or risk factor diagnosis and care in country-level TB guidelines is unknown. In addition, post-tuberculosis lung disease (PTLD), or the “evidence of chronic respiratory abnormality, with or without symptoms, attributable at least in part to previous tuberculosis,” [23] is also increasingly recognized as a significant issue for individuals treated for TB, contributing to increased morbidity and mortality in the post-treatment period [24–26] and the long-term adverse clinical and socioeconomic impacts of TB [27]. Clinical standards and inclusion of PTLD diagnosis and management in TB guidelines have been recommended by TB experts [28,29], but WHO guidelines on PTLD are not currently available and adoption of this recommendation by NTPs is unknown.

Therefore, our scoping review aimed to analyze the variations in how countries with the highest burdens of TB across the six WHO regions approach the integration of comorbidity and risk factor screening, diagnosis and treatment, recurrent TB, as well as the adoption of PTLD diagnosis and management, within their TB guidelines. We also provide recommendations and suggest areas of improvement based on our findings.

## Methods

We used the Arksey and O’Malley methodological framework to perform a scoping review of TB guidelines [30]. The review framework included identifying the research question, searching and selecting relevant guidelines, charting the data, and collating, summarizing, and reporting the results. The review adhered to the PRISMA ScR guidelines [31] (S1 PRISMA Checklist).

### Research question

How do countries with the highest TB, TB-HIV, and MDR-TB burdens approach diagnosis and caring for comorbidities or clinical risk factors among adults being treated for TB and individuals who complete TB treatment but return with symptoms or have evidence of PTLD?

### Search strategy and selection

We identified the 2021–2025 WHO list of 49 countries with the highest TB burden across WHO Regions [32]. Thereafter, we searched through government portals and websites to locate these countries’ most recent national adult TB patient management guidelines up until July 2024, when we froze data collection (S1 Text). In cases where we could not find the guidelines or where the guidelines were outdated, we contacted NTP colleagues whom we knew and/or National TB Program Managers listed on a CDC website (S1 Table) via email or LinkedIn. We categorized guidelines by whether they addressed drug-susceptible (DS) and drug-resistant (DR) TB individually or if they were consolidated. For guidelines not available in English, methods of translation are included in Table 1. Chinese and Kyrgyz translations were not fully interpretable and guidelines from Angola were missing multiple pages so were excluded from analysis.

**Table 1:** WHO list of highest TB burden country descriptions and guideline details.

Country	WHO Region	WHO Classification			TB Guideline Name, Edition, Date	DS*, DR, Consolidated	Primary language and translation source
**TB**	**TB-HIV**	**MDR/RR**
Bangladesh	South-East Asian Region	X		X	National Guideline and Operational Manual for Tuberculosis, Sixth Edition, October 2021	DS	English
Botswana	African Region		X		National Tuberculosis Programme Manual, Eighth Edition, 2017	DS	English
Brazil	Region of the Americas	X	X		Manual of Recommendations for TB Control in Brazil, 2^nd^ Updated Edition, 2019	DS	Portuguese; Translated with DeepL Advanced Pro and CK
Cameroon	African Region		X		Technical Guide for Cameroon’s Healthcare Workers, January 2019	DS	French; Translated with DeepL Advanced Pro
Central African Republic	African Region	X	X		Guide to the Management of Adult Tuberculosis, Fifth Edition, April 2021	DS	French; Translated with DeepL Advanced Pro
Republic of Congo	African Region	X	X		A Guide to the Management of Tuberculosis and HIV/AIDS Co-Infection and the Concept of the One Stop Shop, January 2020 Edition	DS	French; Translated with DeepL Advanced Pro
Democratic Republic of Congo	African Region	X	X	X	Tuberculosis Management Guidelines, Part 6, 2021 Edition	DS	French; Translated with DeepL Advanced Pro
Eswatini	African Region		X		National Tuberculosis Programme Manual, Second Edition, 20 February 2012	DS	English
					National Drug-Resistant Tuberculosis Management Guidelines, Second Edition, 11 September 2012	DR	English
Ethiopia	African Region	X	X		National Guidelines for TB, DR-TB and Leprosy in Ethiopia, 6th Edition	Consolidated	English
Guinea	African Region		X		Technical Guide for Healthcare Staff, 3rd Edition, 2015	DS	French; Translated with DeepL Advanced Pro
					Technical Guide for the Management of Multi-Drug-Resistant Tuberculosis, 3rd Edition, September 2015	DR	French; Translated with DeepL Advanced Pro
India	South-East Asian Region	X	X	X	Technical and Operational Guidelines for Tuberculosis Control in India, 2016	DS	English
					Guidelines for Programmatic Management of Drug-Resistant Tuberculosis in India, March 2021	DR	English
Kenya	African Region	X	X		Integrated Guideline for Tuberculosis, Leprosy, and Lung Disease, 2021	Consolidated	English
Lesotho	African Region	X	X		National Guidelines for Drug Susceptible Tuberculosis, 2019 Edition	DS	English
Malawi	African Region	X			National Tuberculosis and Leprosy Guidelines, Ninth Edition, June 2024	Consolidated	English
Mozambique	African Region	X	X	X	Evaluation and Management of Patients with Tuberculosis, National Protocols, 2019	DS	Portuguese; Translated by CK and DeepL Advanced Pro
					Manual for the Clinical and Programmatic Management of Multidrug- Resistant Tuberculosis, September 2019	DR	Portuguese; Translated by CK and DeepL Advanced Pro
Myanmar	South-East Asian Region	X	X	X	Guideline for Drug Sensitive TB Management in Myanmar, July 2020	DS	English
					Guidelines for the Management of Drug-Resistant TB (DR-TB) in Myanmar, February 2017	DR	English
Namibia	African Region	X	X		National Guidelines for the Management of Tuberculosis, Fourth Edition, March 2019	Consolidated	English
Nepal	South-East Asian Region			X	National Tuberculosis Management Guidelines, 2019	DS	English
					National Guidelines on Drug Resistant Tuberculosis Management, 2019	DR	English
Nigeria	African Region	X	X	X	National Tuberculosis, Leprosy and Buruli ulcer Management and control Guidelines, Sixth Edition, 2015	DS	English
Pakistan	Eastern Mediterranean Region	X		X	National Guidelines for the Control of Tuberculosis in Pakistan, 2019	DS	English
					National Guidelines for the Pragmatic Management of Drug-Resistant Tuberculosis (PMDT), 2020 Edition	DR	English
Papua New Guinea	Western Pacific Region	X		X	National Tuberculosis Management Protocol, 2011	DS	English
Peru	Region of the Americas			X	Technical Health Standard for the Comprehensive Care of the Person Affected by Tuberculosis, Family and Community, 1st Edition, June 2023	DS	Spanish; Translated with DeepL Advanced Pro
Philippines	Western Pacific Region	X	X	X	Manual of Procedures of the National Tuberculosis Control Program, 5th Edition, 2014	DS	English
Republic of Moldova	European Region			X	Tuberculosis in Adults National Clinical Protocol, 2020	Consolidated	Romanian; Translated with DeepL
Sierra Leone	African Region	X			Guidelines for Clinical and Programmatic Management of TB in Serra Leone, March 2024	Consolidated	English
Somalia	African Region			X	Guideline for Drug-Susceptible Tuberculosis, 2022	DS	English
					Management of Drug-resistant Tuberculosis (DR-TB) for Somalia, December 2020	DR	English
South Africa	African Region	X	X	X	National Tuberculosis Management Guidelines, 2014	DS	English
					Management of Drug-resistant Tuberculosis, Policy Guidelines, 2013	DR	English
Thailand	South-East Asian Region	X	X		National Tuberculosis Control Programme Guideline, Thailand, 2018	DS	Thai; Machine Translated by Google and verified by KKKH.
Uganda	African Region	X	X		Manual for Management and Control of Tuberculosis and Leprosy, 3^rd^ Edition, March 2017	DS	English
Ukraine	European Region			X	TUBERCULOSIS Evidence-based clinical guidelines, January 2023	Consolidated	Ukrainian; Translated with DeepL Advanced Pro
United Republic of Tanzania	African Region	X	X		Manual for Management of Tuberculosis and Leprosy in Tanzania, 7th Edition, April 2020	Consolidated	English
Vietnam	South-East Asian Region	X		X	Diagnosis and Treatment Guidelines and Tuberculosis Prevention, March 2020	DS	Vietnamese; Machine translated by Google
Zambia	African Region	X	X	X	National Tuberculosis and Leprosy Programme Consolidated Tuberculosis Guidelines, First Edition, 2022	Consolidated	English
Zimbabwe	African Region		X	X	Zimbabwe National TB Guidelines, June 2018	Consolidated	English
**Country**	**WHO Region**	**WHO Classification**	**Reason for Exclusion**		
		**TB**	**TB-HIV**	**MDR/RR**			
Angola	African Region	X		X	Multiple pages were missing.		
Azerbaijan	European Region			X	Attempts to retrieve guidelines were unsuccessful.		
Belarus	European Region			X	Attempts to retrieve guidelines were unsuccessful.		
China	Western Pacific Region	X	X	X	Translations were not fully interpretable.		
Democratic People’s Republic of Korea	South-East Asian Region	X		X	Attempts to retrieve guidelines were unsuccessful.		
Gabon	African Region	X	X		Attempts to retrieve guidelines were unsuccessful.		
Guinea-Bissau	African Region		X		Attempts to retrieve guidelines were unsuccessful.		
Indonesia	South-East Asian Region	X	X	X	Attempts to retrieve guidelines were unsuccessful.		
Kazakhstan	European Region			X	Attempts to retrieve guidelines were unsuccessful.		
Kyrgyzstan	European Region			X	Translations were not fully interpretable.		
Liberia	African Region	X	X		Attempts to retrieve guidelines were unsuccessful.		
Mongolia	Western Pacific Region	X		X	Attempts to retrieve guidelines were unsuccessful.		
Russian Federation	European Region		X	X	Attempts to retrieve guidelines were unsuccessful.		
Tajikistan	European Region			X	Attempts to retrieve guidelines were unsuccessful.		
Uzbekistan	European Region			X	Attempts to retrieve guidelines were unsuccessful.		

*Note: Guidelines designated as DS without a complementary DR guideline generally included short sections addressing DR TB, but these sections were minimal as compared to consolidated guidelines.

### Charting, collating, summarizing, and reporting the data

A primary investigator (MSB) charted, collated, and summarized the data in a data extraction tool adapted from the Cochrane Effective Practice and Organization of Care group [33]. Secondary investigators (NN, JM, KKKH, CK) reviewed and cross-validated findings from one-third of guidelines. For each guideline, we extracted data on date, edition, DS, DR, or consolidated guidelines and pre-determined key comorbidities and factors that have been shown to be associated with increased TB risk, are common among people with TB disease, have been associated with delayed diagnosis, increased risk of treatment interruption, poorer adherence, higher rates of treatment failure, relapse, and mortality, and/or have been specified in WHO publications [12,34–40] (S1 Data). This included HIV, pregnancy, underlying liver disease, kidney disease, tobacco use, alcohol use, drug/substance use, mental health, diabetes, nutrition, and air pollution [41–51]. We classified each as ‘not mentioned,’ ‘mentioned without recommendations,’ or ‘mentioned with recommendations,’ paying specific attention to whether each was included in guidelines with specific recommendations regarding screening or diagnosis, TB medication regimen adjustments, and/or linkage to additional care specific to the comorbidity or risk factor. We also extracted data on recommendations for end-of-treatment assessment or follow-up, assessments for recurrent TB, and PTLD. Other post-TB morbidities were not included in our data extraction as there is currently limited consensus guidance on how to assess for or manage other post-TB complications such as cardiovascular or neurological sequelae. In countries whose adult and pediatric guidelines were combined, we focused on the sections pertaining to adults.

We iteratively reviewed the data extraction tool and guidelines to aggregate and describe findings. Our findings are reported by select phases of the TB care cascade: 1) during treatment with a focus on screening, TB regimen adjustments and linkage to care, 2) at treatment completion, and 3) post-TB treatment [52] at the country level. Therefore, if a country had separate guidelines for DS and DR TB, and only one of them included screening for specific risk factors or comorbidities, we included that in our count. If both mentioned screening for the same risk factors or comorbidities, we aggregated those findings and reported them as a single result.

## Results

**Fig 1:**
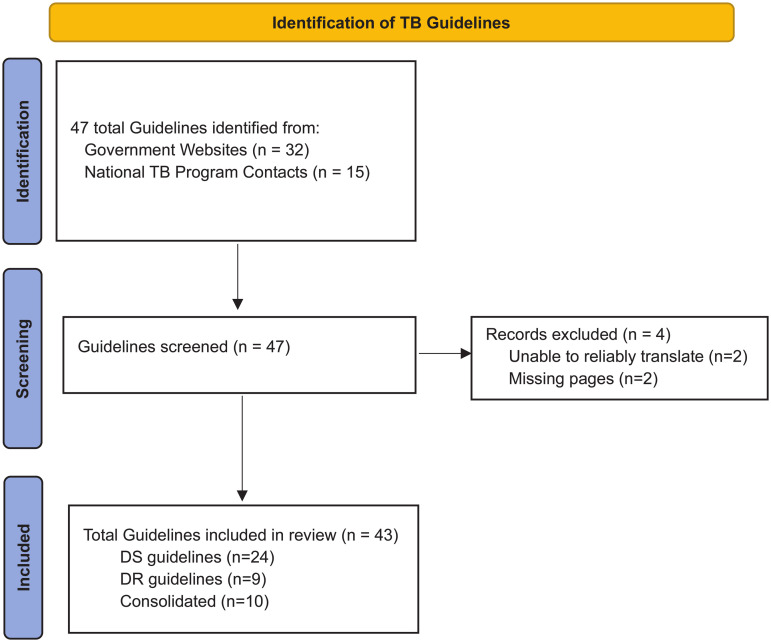
PRISMA flow diagram.

Of the 49 countries with the highest TB burden, we reliably found, translated, and analyzed 43 guidelines from 34 countries (Fig 1). The guidelines included DS (n = 24), DR (n = 9), and consolidated/integrated DS and DR TB disease guidelines (n = 10). Of the 30 highest MDR-TB burden countries, we were able to find and analyze 11 DR or consolidated guidelines. Guideline publication year ranged from 2011 (Papua New Guinea) to June 2024 (Malawi).

## Screening for Co-morbidities and clinical risk factors and TB regimen adjustment

### HIV/AIDS

**Fig 2:**
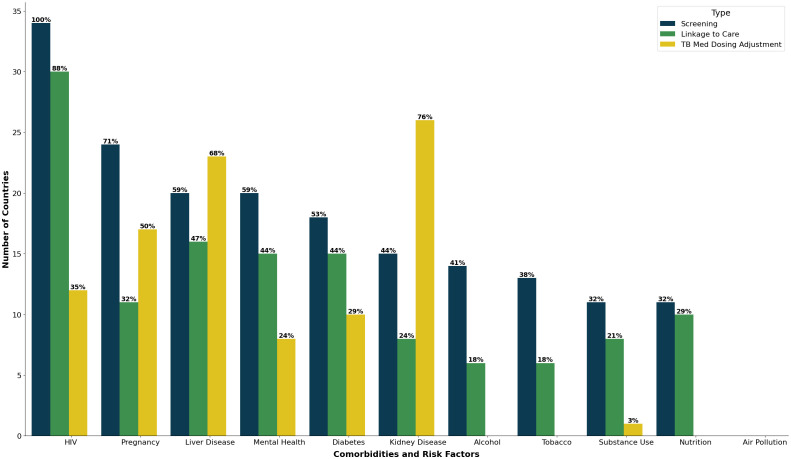
Frequency of recommendation types by comorbidity or risk factor among TB guidelines for 34 countries.

The most recommended comorbidity screening among the guidelines we reviewed was HIV/AIDS, with all 34 (100%) countries mentioning the need to screen for HIV among people with TB (Fig 2). Some countries, such as Namibia and Mozambique, took a bidirectional approach to detecting and diagnosing TB/HIV, where they encouraged providers to screen for TB in HIV clinics and vice versa [53,54]. Others recommended performing provider-initiated HIV testing and counseling in all individuals suspected to have TB [55–57]. In addition to offering HIV testing and counseling to individuals diagnosed with TB, Pakistan and the Central African Republic recommended extending such services to the partners of known HIV-positive individuals with TB [58,59]. Three countries (Peru, Brazil, and Pakistan) specified a screening tool, with Peru recommending using either Enzyme-linked Immunosorbent Assay (ELISA) or Rapid Diagnostic Test (RDT), while Brazil and Pakistan mentioning RDT [58,60,61].

In terms of drug regimen adjustment, most recommendations were in relation to HIV drugs, not TB medications. Botswana had extensive guidelines around HIV and ART regimens for patients with TB that are well aligned with the WHO recommendations [62]. Meanwhile, other countries such as the United Republic of Tanzania, Lesotho, and Nepal specified that individuals infected with HIV with TB disease should receive the same dosages and regimens of anti-TB therapy as people who are not infected with HIV [63–66].

### Pregnancy

The second most recommended screening assessment was pregnancy testing, with 71% (24/34) of countries recommending screening for pregnancy and/or listing pregnancy as one of the “Special Populations” to consider in the treatment of TB. However, Peru and India were the only countries that specified testing for pregnancy with the beta-human chorionic gonadotropin. All other countries either mentioned the need to ascertain pregnancy or specifically stated the need to ask patients about their pregnancy status without further specification. Furthermore, India recommended that people with DR-TB who are found to be pregnant before treatment initiation or whilst on treatment must be assessed in consultation with a gynecologist or obstetrician, “considering factors such as risks and benefits of DR-TB treatment; severity of DR-TB; gestational age; and potential risk to [fetus].”

Sixteen (47%) countries mentioned TB regimen adjustment, with most advising against using streptomycin because of fetal ototoxicity [55,67–69]. Some guidelines also recommended avoiding ethionamide, injectable agents, and aminoglycosides [70–72].

### Underlying liver and kidney disease

Twenty (59%) countries specified screening for underlying liver disease, with 18 recommending liver function tests (LFTs). Underlying liver disease was among the few comorbidities with explicit recommendations often included regarding TB regimen adjustments, with guidelines recommending that pyrazinamide not be given to individuals with TB and underlying liver disorders due to hepatoxicity [61,64,73–75].

While 29 (85%) countries mentioned renal insufficiency, failure, or disease in their guidelines, only 15 (44%) countries recommended screening for this comorbidity, with 7 countries recommending a serum creatinine test. In addition, 24 (83%) recommended dosage adjustments, with specific recommendations varying by country. For example, the Democratic Republic of Congo advised against giving ethambutol to patients with severe renal insufficiency, while countries like Lesotho and Nigeria suggested reducing the dosage and giving it intermittently to patients in facilities that can closely monitor renal function.

### Mental health

Eighteen (53%) countries recommended screening for mental health and psychological disorders among people being treated for TB. However, only Kenya and South Africa mentioned a specific screening tool, the PHQ-9. Malawi mentioned using a Psychosocial Assessment Form but did not provide further details. Fifteen countries suggested referring those with a positive diagnosis for mental disorders to specialists, which included psychologists, psychiatrists, and social workers. Eight (24%) countries specified adjusting the TB regimen among those with mental health issues, with a focus on closely monitoring cycloserine given its known potential to cause psychological disorders.

### Tobacco, alcohol, and drug/substance use

Thirteen (38%) countries mentioned screening for tobacco use, 6 of which provided details on screening techniques, linkage to care, or TB treatment dosage adjustment. Verbal screening was most often recommended. For example, in South Africa, the recommended screening tool for tobacco use is the 5 A’s, which stand for Ask, Advise, Assess, Assist, and Arrange.

About 41% (14/34) of countries recommended screening for alcohol use either as part of the patient’s social history or medical history. Only 3 countries (Kenya, Namibia, and South Africa) had specific screening tool recommendations. The first two specified using the CAGE screening tool and South Africa recommended using WHO’s Alcohol Use Disorders Identification Test (AUDIT) screening tool [76,77].

Eleven (32%) countries also had recommendations regarding screening for drug/substance use, though the specific sections of the guidelines where these were mentioned varied. Somalia, for example, recommended this within the context of treatment adherence while Kenya included substance dependence alongside mental health assessments more generally. Seven countries had specific recommendations beyond screening.

### Diabetes

Among the 18 (53%) countries that recommended screening for diabetes as part of their TB patient management guidelines, 9 specified fasting blood sugar. Other recommended screening tests included random blood sugar and glycated hemoglobin tests. Myanmar also recommended conducting verbal screening for those with known diabetes status. Other countries did not provide guidance on screening for diabetes among individuals with TB; rather, they recommended screening for TB among individuals with diabetes, and these were not included in our count.

More than half of those countries which recommended screening for diabetes (10/18) also specified drug regimen adjustments among those with diabetes. The Brazilian guidelines recommended switching from oral hypoglycemic agents to insulin since rifampicin interacts with the former, which may lead to a decompensation of diabetes. The Eswatini guidelines provided extensive recommendations on education for patients, glucose monitoring, and regular monitoring.

### Nutrition

Eleven (32%) countries mentioned nutritional assessment in their guidelines. Eswatini’s DS guidelines specified detailed steps for nutritional support to people with TB, which include 7 stages, such as screening (measuring height, weight, and mid-upper arm circumference), admission, provision of nutritional treatment, and health education and follow-up. Furthermore, Bangladesh’s DS guidelines specified that nutritional assessment in people with TB should include clinical, anthropometric, and dietary and laboratory assessments. The clinical evaluations include a nutrition-oriented history and a nutrition-oriented examination. Although India, Myanmar, and South Africa did not specifically recommend screening for nutrition among individuals diagnosed with TB, they suggested providing nutritional support, which can take the form of providing food parcels and should include a source of protein whenever possible.

### Air Pollution

Nepal is the only country that mentioned air pollution/quality in its guidelines, but this was in reference to ensuring good ventilation to reduce TB bacillary burden in the air as a means of TB prevention. Air pollution, in the sense of particulate matter or other vapors, dust, gases, and fumes, was not mentioned in any guidelines.

## Linkage-to-care for co-morbidities and clinical risk factors

Frequency of linkage-to-care recommendation inclusion varied by condition or risk factor: HIV (88%), underlying liver disease (47%), diabetes (44%), pregnancy (32%), drug/substance use (21%), nutrition (29%), tobacco use (18%), alcohol use (18%), mental health (15%), and underlying renal disease (9%). However, very few of these guidelines specified a pathway for linkage to care. For example, while HIV had the highest number of linkage-to-care recommendations, only 3 countries specified a pathway. Details about linkage to care also varied considerably by diagnosis and between the different country guidelines. Examples included Tanzania, which specified co-locating HIV and TB care except when the TB clinic does not provide integrated TB and HIV services (in which case they should be referred to the HIV Care and Treatment Center). Eswatini discussed providing nutritional support to all people with TB and are malnourished when they visit participating health facilities. The Philippines guidelines did not provide a specific linkage-to-care pathway for tobacco use and diabetes but broadly recommended referral to a separate service outside of the TB care system.

## End of treatment assessment

Twenty countries (59%) discussed assessing patients for symptoms or complications after TB treatment completion. Recommended assessments included AFB smears, TB cultures, chest X-rays, and collecting body weight and anthropometric data. In addition to these assessments, Kenya recommended screening for substance abuse as part of the post-treatment evaluations. South Africa also recommended that clinicians trace all failures to attend appointments and highlighted importance of knowing the patients’ residence for follow-up.

Countries varied on the frequency of post-treatment assessments, with some, such as Ukraine and Zimbabwe, noting a one-time assessment, while others, such as India, Myanmar, Nepal, South Africa, and Pakistan, recommended a follow-up period every 6 months for two years in their DR guidelines.

Other countries explicitly stated it was unnecessary to assess individuals after TB treatment completion. Pakistan, for example, noted in its drug-sensitive guidelines that it is unnecessary to follow patients who successfully completed their TB treatment but mentioned that these individuals should be asked to report for re-examination if symptoms recur. The Central African Republic (CAR) also indicated that clinical monitoring post-treatment is not absolutely essential; though, it emphasized the need to warn patients to return immediately to a Tuberculosis Diagnostic and Treatment Center if they develop any suspicious symptoms. Further, for people living with HIV, the CAR recommended prolonged monitoring due to their heightened risk of relapse and reinfection.

## Post-TB treatment

### Recurrent TB Diagnostics and Non-TB investigations in the returning patient

Twenty-seven (79%) countries specified diagnostic tests assessing for TB recurrence among individuals returning with symptoms (Table 2). Most recommendations were in the context of considering DR-TB in a previously treated patient, rather than a separate discussion of how to diagnose recurrent TB. GeneXpert Mycobacterium tuberculosis and rifampicin (MTB/RIF) was the most common screening technique suggested (25/27; 93%). Fourteen of the 27 countries (52%) recommended culture with drug sensitivity testing (DST), while other diagnostics included Line Probe Assay (LPA) and sputum microscopy.

**Table 2:** Specific diagnostic recommendations among 27 countries which mentioned recurrent TB diagnosis among individuals with DS-TB in guidelines.

Country					
	AFB	Culture and DST	LPA	TSA	Xpert MTB/Rif
Bangladesh		X			
Brazil^a^	X	X			X
Botswana		X			X
Central African Republic		X			X
Democratic Republic of Congo					X
Eswatini			X		X
Ethiopia					X
Guinea^b^		X			X
Kenya		X			X
Lesotho		X	X		X
Malawi			X		X
Mozambique^c^		X	X	X	X
Myanmar			X		X
Nepal		X	X		X
Pakistan					X
Papua New Guinea		X			
Philippines					X
Republic of Moldova					X
Sierra Leone			X		X
Somalia^d^			X		X
South Africa		X	X		X
Thailand	X	X	X		X
Uganda					X
Ukraine^e^					X
United Republic of Tanzania		X			X
Vietnam					X
Zambia		X	X		X

Legend: (abbv): AFB/BAAR = acid fast bacilli or acid-alcohol resistant bacilli; MTB/Rif = Mycobacterium Tuberculosis and Rifampicin; LPA = Line Probe Assay; DST = Drug Susceptibility Testing; TSA = Tuberculostearic Acid

^a^Brazil also recommended chest x-ray and computed tomography of chest

^b^The Guinean DS guidelines recommended antibiogram alongside Xpert MTB/RIF and culture. Also stated that patients whose sputum is positive on direct examination after retreatment are usually carriers of bacilli resistant to several antibiotics and should be referred to specialized centers where they can undergo culture and sensitivity testing to determine the appropriate treatment.

^c^Mozambique guidelines recommended requesting a test to quickly determine the existence of resistant TB (Xpert MTB/RIF, LPA), in addition to culture and TSA.

^d^The Somali DS guidelines stated that all individuals who were previously treated for TB should have access to molecular testing (Xpert MTB/RIF, Ultra assays, Truenat, LPA) before initiating treatment to determine the correct treatment regimen.

^e^Ukraine guidelines specify that this is a conditional recommendation, with low quality of evidence for accuracy: in adults with symptoms of pulmonary TB and a history of TB treatment completed within the last 5 years, Xpert Ultra can be the primary diagnostic test for TB and for detecting rifampicin resistance in sputum, instead of smear microscopy, culture, and DST.

None of the countries in our review mentioned conducting non-TB investigations for patients previously treated for TB who return with respiratory or constitutional symptoms, though some did highlight non-TB complications. Guinea Conakry recommended doing an exhaustive clinical review and examination of all body systems, including cardiovascular, digestive, locomotor, urogenital, and nervous systems. Brazil mentioned considering TB sequelae when evaluating diagnostic imaging for TB, Botswana specified entertaining alternate diagnoses to TB including bacterial pneumonia, silicosis, and persistent lung damage from prior TB, and Republic of Moldova included a differential diagnosis for pulmonary TB including lung cancer, bronchiectasis, suppurative lung disease, and pneumonia.

### Post-TB lung disease

Only 7 (21%) countries (Democratic Republic of Congo, Kenya, Malawi, Peru, South Africa, Uganda, and Zambia) mentioned post-TB lung disease in their guidelines (Table 3). The Democratic Republic of Congo recommended that individuals be examined for residual respiratory symptoms and that those with post-TB respiratory pathology should be referred to a doctor for further evaluation. The guidelines also mentioned that chest X-ray may be helpful to assess post-TB sequelae.

**Table 3:** Specific recommendations among countries which mentioned ptld in guidelines.

Country	Assessment	PTLD Manifestation	Management and Referral
**Democratic Republic of Congo**	Recommended screening for residual respiratory symptoms (shortness of breath, wheezing, coughing up sputum, etc.) Noted that CXR may help identify post-TB sequelae.	Not specified.	Refer to doctor for post-tuberculosis respiratory pathology.
**Kenya**	Baseline assessment at treatment start: document signs, symptoms, CXR, diagnostic tests. During treatment: monitor symptoms, signs, smear microscopy. Post-treatment: CXR (6 months for DS-TB, end of treatment for DR-TB), clinical exam at 6 months, counseling on PTLD symptoms.	Specified investigations to specifically assess for fibrosis, bronchiectasis, COPD, lung abscess, aspergillus-related lung disease, spontaneous pneumothorax.	Management varies by diagnosis (e.g., abscess treated with antibiotics guided by culture). Also recommended referral to chest physician after PTLD diagnosis.
**Malawi**	Specified 4 diagnostic criteria: persistent respiratory symptoms, lung function abnormalities (spirometry), CXR, reduced exercise tolerance.	Not specified.	Pulmonary rehabilitation and other medical management such as corticosteroids to reduce inflammation and alleviate symptoms in cases of inflammation and airway narrowing.
**Peru**	Recommended adequate referral of the case once the patient is discharged from the TB program to continue care and follow-up as appropriate by the destination service and to carry out the respective monitoring of the compliance and progress.	Divided into: Structural - bronchiectasis, destroyed lung, cavities, trapped lung, bronchopleural fistula. Functional - respiratory insufficiency, bronchial hyperreactivity.	Respiratory rehabilitation, which includes strengthening of respiratory muscles and general physical retraining as well as controlled breathing techniques.
**South Africa**	Not specified.	DR-TB guidelines specified that residual lung disease, shortness of breath, cough is common, especially in DR-TB patients with extensive lung damage.	Use of bronchodilators after treatment completion; ancillary meds to reduce morbidity and mortality; monitoring of treatment response.
**Uganda**	Repeat TB evaluation if symptoms recur; If negative, assess for other conditions No specific tests or procedures specified.	Recurrent post-TB symptoms; bronchiectasis, COPD, pulmonary hypertension, colonization with fungal infections such as aspergillosis.	Not specified.
**Zambia**	Caution in interpreting CXR in previously treated TB patients when TB tests are negative.	Not specified.	Not specified.

COPD: chronic obstructive pulmonary disease; CXR: chest x-ray; DS-TB: drug-sensitive tuberculosis; DR-TB: drug-resistant tuberculosis

Kenya recommended assessing for PTLD starting with a baseline assessment at the beginning of pulmonary TB treatment followed by periodic assessments during and after successful completion of pulmonary TB treatment, as outlined in Table 3. The guidelines specified various investigations to assess PTLD manifestation, such as fibrosis, bronchiectasis, chronic obstructive pulmonary disease (COPD), lung abscess, aspergillus-related lung disease, and spontaneous pneumothorax and provided management strategies for these conditions. Once a PTLD diagnosis is established, the guidelines recommended referral to a chest physician for further specialized care.

Malawi specified 4 diagnostic criteria for PTLD: persistent respiratory symptoms, abnormalities in lung function tests (spirometry), abnormalities on chest X-ray, and reduced exercise tolerance. It also provided some recommendations for managing PTLD, including pulmonary rehabilitation and medical management. Peru’s guidelines discussed monitoring persons with TB for the identification of possible complications, damage, or sequelae and divided pulmonary sequelae into structural and functional impairments. They recommended ensuring adequate referral after discharge from the NTP and discussed the importance of respiratory rehabilitation, specified as including strengthening of respiratory muscles and general physical retraining as well as controlled breathing techniques.

The South African DR-TB guidelines discussed the high likelihood of extensive lung damage in individuals with MDR-TB and had a section on recommended ancillary medications which can reduce morbidity and mortality and improve overall treatment outcomes in individuals with DR-TB. They specifically noted that bronchodilators can alleviate shortness of breath and suppress cough and recommended that “due to the high prevalence of residual lung disease in [people with DR-TB], bronchodilators should be continued after completion of treatment.”

In Uganda, individuals who present post-TB with recurrent symptoms are acknowledged as a common and important population to consider. They underscored the lack of global standard guidelines on PTLD evaluation but emphasized a repeat evaluation for TB and if negative, further re-evaluation for specific processes such as bronchiectasis, COPD, pulmonary hypertension, and colonization with fungal infections. However, no tests or procedures were specified for this evaluation. Finally, Zambia mentioned PTLD solely in the context of cautioning that care must be taken when interpreting CXRs of patients with a prior history of TB when repeat TB diagnostic testing is negative given that patients with a prior history of TB may present with symptoms suggestive of TB due to PTLD.

## Discussion

This review examined the different approaches countries take in addressing TB-comorbidity and risk factor screening and management, end-of-TB and post-TB assessments, and PTLD in their national TB guidelines. Our findings highlight significant variability in guideline recommendations across 34 of the highest TB-burden countries and underscore both strengths and gaps in current TB guidelines and recommendations. There are limitations in how screening and linkage to care for comorbidities and risk factors beyond HIV are addressed, how to investigate recurrent TB symptoms post treatment completion, and how to assess for PTLD and provide additional support for patients. In highlighting these limitations in the context of current evidence and global guidance, this review has important implications for future guideline development, TB research and policy development.

Our work builds on prior work by Jarda et al. who conducted a survey of TB providers across 27 high-TB burden countries and found that while most TB programs screened and managed HIV, fewer than half screened or treated for diabetes, tobacco and alcohol use disorders, and almost none screened or managed mental health. [78]. Our study adds to this work by directly reviewing country guidelines and examining PTLD. We similarly found that all TB guidelines addressed HIV, but substantially fewer addressed mental health, diabetes, tobacco and alcohol use. Indeed, many guidelines described bidirectional approaches for TB and HIV detection, and nearly all dedicated specific sections or chapters to HIV/TB collaborative service. This was juxtaposed to how guidelines addressed mental health, nutrition, diabetes, liver or kidney disease, tobacco, alcohol, and substance use, most of which were mentioned in less than half of country guidelines. Recommendations that were made often lacked specificity to help guide providers on how best to screen, diagnose, and link patients to care despite their serious impacts on treatment outcomes. This highlights an important opportunity for future iterations of both WHO-level and country-level guidelines.

WHO, TB experts, and public health leaders have emphasized the importance of addressing multimorbidity, ideally through integrated health services, in the effort to eradicate TB (75–77). TB-HIV guidelines and sections can serve as a model for how to address other prevalent TB comorbidities and risk factors and be a source of strategies for overcoming implementation barriers for integration strategies and interventions, such as limited patient and provider knowledge and awareness of comorbid conditions, lack of resources or programmatic funding, increased workload to NTP providers, and stigma (81–83). Furthermore, the WHO TB comorbidity guidelines should serve as a key resource for country-level guideline development and can be referenced directly. Their emphasis on the importance of integrated care and example models of integrated mental health-TB, HIV-TB and diabetes-TB services may further inform future iterations of country-level guidelines. Hopefully, future iterations of WHO comorbidity guidelines will continue to model how countries can integrate services, such as smoking cessation or PTLD screening, under chronic disease management or lung health to improve implementation.

Given the extensive processes and time required to finalize WHO and country-level guidelines, NTPs may also benefit from learning from one another’s approaches to these complex questions. For example, Eswatini’s guidelines provide a great example for nutritional assessment and support which could be modelled – they reference their Food by Prescription program, a joint initiative of the National TB Control Program, the Swaziland Nutritional Council, the World Food Programme, WHO, and other partners, which provides food by prescription to people with TB [79]. Launching similar initiatives in high-TB-burden countries, and highlighting such resources in treatment guidelines, could be a significant step in preventing and mitigating the adverse effects of malnutrition and TB.

Another opportunity for structured guidance is around recurrent TB diagnostics and non-TB investigations in individuals treated for TB who return with symptoms. This is especially notable given the high rates of TB recurrence in high-TB burden countries and that previous TB remains a strong risk factor for incident TB disease, either due to treatment failure and relapse, or because people return to similar social environments with ongoing risk factors that have not been addressed, thereby yielding high rates of reinfection [80]. About 80% of countries provided recurrent TB diagnostic recommendations, with most recommending Xpert MTB/RIF. Yet, specificity of this test is reduced amongst those previously treated for TB due to the potential presence of residual DNA from past infections [81,82]. While TB culture is the reference standard for TB diagnosis, the availability and quality of culture in many high TB burden settings is limited [83–85]. Thus, the potential for false positive Xpert MTB/RIF results in the absence of culture may lead to unnecessary TB treatment and should be highlighted in country-level guidelines. Furthermore, none of the guidelines included in this review mentioned testing for non-Mycobacterium tuberculosis (Mtb) infection (e.g., other bacteria, viral, or fungal infections) in patients who have been treated for TB at least once and are returning with respiratory or constitutional symptoms. This is despite data that secondary pulmonary infections or co-infections with organisms such as Aspergillus or non-TB mycobacterium (NTM) are increased among individuals with prior TB as compared to those whom are not previously been diagnosed with TB [86,87]. We believe the lack of NTM infection diagnoses could be a result of limited awareness among healthcare providers and inadequate laboratory resources for identification and speciation in these high TB-burden countries [88].

End-of-treatment and post-treatment assessments and care were also not frequently addressed in the guidelines. Only 7 countries explicitly addressed PTLD, signaling a critical gap between expert recommendations and WHO or national-level implementation. While our review encompasses over 13 years of guidelines, even among the 28 guidelines published since 2019, 75% had no guidance on assessing and managing PTLD despite its significant long-term negative clinical and socioeconomic effects on TB survivors. Among those that did, Kenya had the most comprehensive overview and may serve as a model for other countries. The remaining 6 countries provided little direction on how to screen, diagnose, and/or manage post-TB lung disease. This admittedly is difficult to do given the limited existing data on these areas, lack of guidance by the WHO, and ongoing debates on whether post-TB care falls within the remit of NTPs. Yet, recognition of PTLD as an important consequence of TB and contributor to increased mortality among TB survivors and context-specific recommendations for referral or linkage to care may be helpful to specify within guidelines. Emerging recommendations from international societies such as the Brazil Thoracic Society and Latin American Thoracic Society may serve as important resources for NTPs in this regard [89,90].

The strengths of this scoping review include its comprehensive evaluation of TB management guidelines from multiple high-burden countries across the six WHO regions, the multidisciplinary and multilinguistic research team which allowed for wider inclusion of guidelines across regions, and examination of DS, DR, and consolidated guidelines. Limitations of the study include difficulty obtaining TB patient management guidelines across all 49 countries with the highest TB burden, thus limiting generalizability. This was largely due to a lack of publicly available guidelines as well as a lack of reliable translations. The wide range of publication dates of guidelines, spanning 13 years, may also not reflect current practices and approaches in many countries. Finally, we did not systematically collect mentions of other post-TB sequelae beyond PTLD such as cardiac or neurological sequelae, which is an important focus for future work.

## Conclusion

This review assessed the country-level gaps and variations in guidelines for the management of TB across the six WHO regions in their integration of TB risk factors, comorbidities, TB recurrence, and PTLD screening, diagnosis, and treatment. Given that national TB guidelines often go through periodic revisions to reflect new scientific findings, advancements in medical technology, and a shift in the epidemiological landscape, guideline developers and NTP leadership should consider how they might provide more specificity around comorbidity and risk factor screening, diagnosis, and treatment, along with the integration of PTLD diagnosis and treatment in subsequent iterations of country guidelines.

## Supporting information

S1 PRISMA Checklist Preferred Reporting Items for Systematic reviews and Meta-Analyses extension for Scoping Reviews (PRISMA-ScR) Checklist. (PDF)

S1 Text Literature Search for Ministry of Health Tuberculosis Guidelines. (DOCX)

S1 Table List of National TB Program Contacts. (PDF)

S1 Data Data Extraction Tool with Primary Data. (XLSX)
