# Supplementary material for: Assessment of comorbidities, risk factors, and post tuberculosis lung disease in National Tuberculosis Guidelines: A scoping review
Source: PLOS Glob Public Health. 2025 Jul 23;5(7):e0004935. doi: 10.1371/journal.pgph.0004935 (PMC12286338; doi:10.1371/journal.pgph.0004935)
Supplement: S1 Text — (DOCX) [file pgph.0004935.s002.docx]

**Supplementary File:**

**Literature Search for Ministry of Health Tuberculosis Guidelines**

A systematic search was conducted to identify national tuberculosis (TB) guidelines issued by Ministries of Health for the 49 countries prioritized for this analysis. The search focused on capturing the most recent TB guidelines. MEDLINE/PubMed and the International Guideline Library were searched using the keywords “tuberculosis” or “tb” when no guidelines were identified. We then searched the grey literature. Sources included official government and Ministry of Health websites, national tuberculosis program (NTP) portals, public health agency websites, and international health organization repositories (e.g., WHO, Stop TB Partnership).

The search strategy involved:

- Direct review of Ministry of Health and national TB program websites for available guidelines, policies, protocols, and strategic plans related to tuberculosis diagnosis, treatment, and management.
- Use of search engines (e.g., Google) with targeted search terms combining country names with keywords such as “tuberculosis guidelines,” “TB protocols,” “national TB program,” “TB policies,” and “Ministry of Health.”
- Review of bibliographies from recent systematic reviews, technical reports, and publications to identify additional guideline documents.

Documents were included if they were published or endorsed by a Ministry of Health or national TB program, represented formal guidance or policy related to any aspect of tuberculosis care or control, and were publicly accessible. Non-English documents were translated as necessary using translation tools such as DeepL and Machine Translation by Google and/or language experts. The search was conducted between June 01, 2023, and October 27, 2024, to ensure inclusion of the most up-to-date guidance.
