# Supplementary material for: Assessment of comorbidities, risk factors, and post tuberculosis lung disease in National Tuberculosis Guidelines: A scoping review
Source: PLOS Glob Public Health. 2025 Jul 23;5(7):e0004935. doi: 10.1371/journal.pgph.0004935 (PMC12286338; doi:10.1371/journal.pgph.0004935)
Supplement: S1 Table — (PDF) [file pgph.0004935.s003.pdf]

Country	NTP Manager	Title	E-mail
**Afghanistan**	Dr. Mohmmad Khaled Seddiq	NTP Manager	mkhaledseddiq@gmail.com
**Albania**	Donika Mema	Manager National Program Tuberculosis	bardhidonika@yahoo.com
**Algeria**	SOFIANE ALIHALASSA	National Tuberculosis Program Manager	alihalassa25@gmail.com
**Andorra**	Clara Palma Jordana	Técnico en Salud Pública	appvs@govern.ad
**Angola**	Ambrosio DISADIDI	Coordenador do Programa Nacional<br>de Controlo da Tuberculose	adisadidi@gmail.com
**Anguilla**	Aisha Andrewin	Chief Medical Officer	aisha.andreiwn@gov.ai
**Argentina**	Marcela Natiello	Coordinadora	marcelanatiello.tbc@gmail.com
**Armenia**	Lusine Kocharyan	NTC Director	kocharyan_lus@yahoo.com
**Aruba**	Wilmer Salazar	MD; medical advisor<br>Dept. Public Health Aruba	wilmer.salazar@despa.gov.aw
**Australia**	National Tuberculosis<br>Advisory Committee	Australian Government<br>Department of Health	Tbdata@health.gov.au<br>ntac.secretariat@health.gov.au
**Azerbaijan**	Viktor Gasimov	NTP manager	viktor.qasimov@health.gov.az
**Bahamas**	JACQUELYN NEWBOLD	TB -PROGRAMME -COORDINATOR	JACQUELINENEWBOLD@BAHAMAS.GOV.BS
**Bahrain**	Ebrahim AlRomaihi	Consultant Internist & NTP Manager	eromaihi@health.gov.bh
**Bangladesh**	Md. Shamiul Islam	Director MBDC &<br>Line Director TB, Leprosy & ASP	directormbdc@gmail.com
**Barbados**	RAJAMANICKAM MANOHAR SINGH	NATIONAL TB CONTROL OFFICER	manohar.singh@barbados.gov.bb<br>drramanoharsingh@gmail.com
**Belarus**	Геннадий Гуревич	Директор ГУ<br>"РНПЦ пульмонологии и фтизиатрии"	ge.gurev@gmail.com
**Belize**	Francis Morey	Deputy Director of Health Services	fmorey@health.gov.bz
**Benin**	AFFOLABI Dissou	Professeur	affolabi_dissou@yahoo.fr
**Bermuda**	Dr. Cheryl Peek-Ball	Chief Medical Officer	cepeekball@gov.bm
**Bhutan**	Ugyen Dendup	Senior Programme Officer	udendup@health.gov.bt
**Bolivia<br>(Plurinational State of)**	Carmen Arraya Gironda	Responsable Programa Nacional<br>de Control de Tuberculosis	carmenarrayag@hotmail.com
**Bosnia and<br>Herzegovina**	Unit for TB control<br>in Bosnia and Herzegovina<br>Hasan Žutić<br>Mladen Duronjić<br>Velimir Bereš	NTP Coordinators of Federation BiH<br>Republic+E12 of Srpska<br>Brčko District of BiH - Members of the Unit<br>for<br>TB control in BiH	h_zutic@bih.net.ba<br>mladenduro@gmail.com<br>velimir.beres@yahoo.com
**Botswana**	Dr Goabaone Rankgoane-Pono	NTP Manager	goaba2000@yahoo.com

**Brazil**	Denise Arakaki-Sanchez	Coordinator,<br>National Tuberculosis Program	denise.arakaki@saude.gov.br
**Brunei Darussalam**	Dr Hjh Anie Haryani Hj Abd Rahman	Director,<br>Environmental Health Services	anie.rahman@moh.gov.bn
**Bulgaria**	Tonka Varleva	Director of Health Promotion Directorate<br>Disease Prevention and Dependence	TVarleva@hdp.bg
**Burkina Faso**	Adjima COMBARY	Coordonnateur	adjicomb@yahoo.fr
**Burundi**	Herménégilde NZIMENYA	Directeur du Programme National Intégré<br>Lèpre et Tuberculose (PNILT)	hermenzi2014@gmail.com
**Cabo Verde**	Jorge Noel Barreto	NTP Manager	drjorgeba@yahoo.com.br
**Cambodia**	Mao Dr. Tan Eang	Director	mao@online.com.kh
**Cameroon**	Vincent MBASSA	NTP Manager	vincentmbassa@yahoo.fr
**Cayman Islands**	Samuel Williams	Medical Officer of Health	samuel.williams@hsa.ky
**Central African Republic**	Hervé Gildas GANDO	Chef de service de lutte<br>contre la tuberculose	gahe_gi@yahoo.fr
**Chad**	Oumar Abdelhadi	Médecin	oumarabdelhadi@yahoo.fr
**Chile**	Nadia Escobar Salinas	Directora Programa Nacional<br>de Tuberculosis	nadia.escobar@minsal.cl
**China**	Jianjun Liu		liujj@chinacdc.cn
**China, Hong Kong SAR**	Chi Kuen Chan	Consultant Chest Physician in-charge	chikuen_chan@dh.gov.hk
**China, Macao SAR**	Mei Jian	Head of Tuberculosis Prevention<br>and Treatment Centre	ctb@ssm.gov.mo
**Colombia**	Julián Trujillo Trujillo	Coordinador Grupo Enfermedades<br>Emergentes,<br>Reemergentes y Desatendidas	jtrujillot@minsalud.gov.co
**Congo**	franck hardain okemba okombi	Coordonnateur du Programme National<br>de Lutte contre la Tuberculose	franckokemba@gmail.com
**Cook Islands**	Josephine Aumea Herman Tepai	Secretary of Health	josephine.herman@cookislands.gov.ck
**Costa Rica**	Aarón Agüero ZumbadoZeidy Mata AzofeifaMinisterio Salud-CajaCostarricense Seguro Social	Coordinador del Ministerio de SaludCoordinadora de la CCSS	aaron.aguero@misalud.go.crzmata@ccss.sa.cr
**Côte d'Ivoire**	Jacquemin KOUAKOU KOUAKOU	Directeur coordonnateur	jacquemink@yahoo.fr

**Cuba**	Suarez Alvarez Lourdes	Coordinadora de Programa Nacional de Control de la Tuberculosis	lourdes.suarez@infomed.sld.cu
**Democratic People's Republic of Korea**	CHOE KUM SONG	National TB Program Manager, Ministry of Public Health	bogon.moph@star-co.net.kp
**Democratic Republic of the Congo**	MICHEL KASWA	DIRECTEUR DU PROGRAMME	mecckay2002@yahoo.fr
**Djibouti**	Hawa Hassan Guessod	Coordinatrice du Programme national de lutte contre la tuberculose	hawahassangue@yahoo.fr
**Dominica**	Shalauddin Ahmed	National Epidemiologist	shalauddin_a@yahoo.com
**Dominican Republic**	Marcelino Belkys	Coordinadora División de Tuberculosis	belkys.marcelino@ministeriodesalud.gob.do
**Ecuador**	Ángela María Sánchez Vélez	Responsable Nacional de la Estrategia de Tuberculosis	angela.sanchez@msp.gob.ec
**Egypt**	Dr. Wagdy Amin	Manager of NTP Egypt	drwagdy@yahoo.com
**El Salvador**	Julio Garay Ramos	Coordinador Programa Nacional de Tuberculosis y Enfermedades Respiratorias	jgaray@salud.gob.sv
**Equatorial Guinea**	JUAN EYENE ACURESILA	DIRECTOR NACIONAL PNLT	pastorredimido@gmail.com
**Eritrea**	Hiwet Nugusse	National TB and Leprosy control program manager	hiwetnb@gmail.com
**Estonia**	Piret Viiklepp	Head of Estonian Tuberculosis Registry	piret.viiklepp@tai.ee
**Eswatini**	Themba Dlamini	Mr	thembatb@yahoo.com
**Ethiopia**	Taye Letta	National TB and Leprosy Programme coordinator	Taye Letta <tayeletta@gmail.com>
**Fiji**	Sam Fullman	Acting National TB Control Officer	sam.e.fullman@gmail.com
**French Polynesia**	GIARD Marine	Médecin responsible du Bureau de veille sanitaire	marine.giard@sante.gov.pf
**Gabon**	MAHOUMBOU Jocelyn	Directeur du Programme National de lutte contre la tuberculose	mahoumboujocelyn@yahoo.fr
**Gambia**	Mr Musa B Jallow	Ag. Programme Manager	musajallo2@gmail.com
**Georgia**	Zaza Avaliani	Director	avalianizaza@yahoo.com
**Germany**	Walter Haas	NTP Manager	HaasW@rki.de
**Ghana**	Dr Yaw Adusi-Poku	Ag Programme Manager	togobay@yahoo.com
**Greenland**	Henrik L. Hansen	Chief Medical Officer	henh@nanoq.gl
**Grenada**	Shawn Charles	Medical Officer of Health, Epidemiology (Ag.)	scharles.moh@outlook.com

**Guam**	Chima Mbakwem	Tuberculosis/Hansen's Disease Control Program Manager	Chima.Mbakwem@dphss.guam.gov
**Guatemala**	Ramirez Sagastume Norma Lucrecia	Coordinadora Programa de Tuberculosis	lucrecia.ramirez@mspas.gob.gt
**Guinea**	ADAMA MARIE BANGOURA	COORDINATRICE NATIONALE	adabangou@yahoo.fr
**Guinea-Bissau**	Miguel Camara	Coordenador de PNLT	miguelcamara68@hotmail.com
**Guyana**	Jeetendra Mohanlall	Manager	jeet21001@gmail.com
**Haiti**	Willy Morose	Coordonnateur National TB	willymorose8@gmail.com
**Honduras**	Artiles Milla Noma Leticia	Coordinadora Nacional de Tuberculosis	normaartiles@yahoo.com
**Hungary**	Gábor Kovács	TB program manager	kovac@koranyi.hu
**India**	Kuldeep Singh Sachdeva	Dr	ddgtb@rntcp.org
**Indonesia**	Imran Pambudi	NTP Manager	imranpambudi@gmail.com
**Iran (Islamic Republic of)**	Nasehi Mahshid	National Mananger of TB Programme	mnasehi@yahoo.com
**Iraq**	Samer Alaubaidy	NTP manager	samertbiq@yahoo.com
**Ireland**	Joan O Donnell	Specialist in Public Health Medicine	joan.odonnell@hse.ie
**Israel**	Dr. Daniel Chemtob	National TB Program Manager and Director, Department of TB and AIDS Ministry of Health, Israel	daniel.chemtob@moh.health.gov.il
**Jamaica**	Nicola Skyers	HIV/STI/TB Director	skyersn@moh.gov.jm
**Jordan**	Ghazi Sharkas	Director of Chest Disease & Foreigners Health Directorate	ghazi.sharkas@moh.gov.jo
**Kazakhstan**	Malik Adenov	Директор Национального научного центра фтизиопульмонологии МЗ РК	malikadenov@mail.ru
**Kenya**	Maureen Kamene	Program Manager	kimaureen@nltp.co.ke
**Kiribati**	Alfred Tonganibeia	National Communicable Disease Specialist	tonganalfredbeia@gmail.com
**Kuwait**	AL Saidi Fatmah	Director ofPulmonary Rehabilitation Center	fatmahalsaidi@hotmail.com
**Kyrgyzstan**	Кадыров Абдуллаат Саматович	директор, Национального центра фтизиатрии	abdylat.kadyrov@gmail.com
**Lao People's Democratic Republic**	Phonenaly CHITTAMANY	Director of National TB Programme	cphonenaly@yahoo.com
**Lebanon**	Yaacoub Hiam	NTP manager	hiamyaacoub@gmail.com

**Lesotho**	LLANG MAAMA	National TB and Leprosy<br>Programme Manager	maama36@hotmail.com
**Liberia**	Roxanne Boker	Program Manager	roxanneboker@yahoo.com
**Luxembourg**	Pierre Weicherding	Médecin chef de Division	pierre.weicherding@ms.etat.lu
**Madagascar**	ANDRIAMAMONJY<br>RAZAFINDRANAIVO<br>Turibio Anderson	Coordonnateur National de lutte<br>contre la Tuberculose	turibioanderson@yahoo.fr
**Malawi**	Mpunga James Upile	Deputy Director,<br>Preventive Health Services	mpungajay@gmail.com
**Malaysia**	Mohamed Naim bin Abdul Kadir	Head of Tuberculosis / Leprosy Sector<br>Ministry of Health	drnaim@moh.gov.my
**Maldives**	Fathaath Hassan	Public health program officer	fathaath@health.gov.mv
**Mali**	Bakary KONATE	Coordinateur du programme	bakarykkonate92@gmail.com
**Malta**	Dr Analita Pace Asciak	Resident Specialist	analita.pace-asciak@gov.mt
**Marshall Islands**	Risa Bukbuk	National TB Program Manager	rmintp@gmail.com
**Mauritania**	Ahmed Tidjane ANNE	COORDINATEUR	ahmedtidjaneanne@yahoo.fr
**Mauritius**	Rujeedawa Mohammed Fezul	Consultant, Chest Diseases<br>TB Program Manager	mrocc@govmu.org
**Mexico**	Luna López Fátima Leticia	Directora de Micobacteriosis	fatima.luna@salud.gob.mx
**Micronesia (Federated States of)**	Ekiek Mayleen Jack	Medical Director,<br>National Communicable Disease Programs	mekiek@fsmhealth.fm
**Mongolia**	Enkhmandakh Danjaad	National TB control programe manager,<br>Head of TB department NCCD	enkhmandakh_0825@yahoo.com
**Montenegro**	Biljana Grbavčević	NTP manager	brezovik@t-com.me
**Montserrat**	Violet Brown	Community Nursing Manager<br>TB Control Officer	brownv@gov.ms
**Morocco**	Laila Bouhamidi	Responsable du Programme National de<br>lutteantituberculeuse	drbouhamidilaila@gmail.com
**Mozambique**	Ivan Manhiça	National TB Program Director	ivanmca2004@yahoo.com.br

**Myanmar**	CHO CHO SAN	Programme Manager, National TB Programme<br>Department of Public Health<br>Ministry of Health and Sports, Myanmar	drchochosanmph@gmail.com
**Namibia**	Albertina Martha Thomas	Chief Health Programme Officer	thomasa@nacop.net
**Nauru**	Jane Short	TB and Leprosy	dowabobojane@yahoo.com
**Nepal**	Dr. Bhim Singh Tinkari	Director, National Tuberculosis Center	ntpdirector@nepalntp.gov.np
**Netherlands**	Gerard de Vries	Coordinator TB Control at RIVM-CIb	gerard.de.vries@rivm.nl
**New Caledonia**	Dominique MEGRAOUA	Medecin	dominique.megraoua@ass.nc
**New Zealand**	Laurence Holding	Manager, Communicable Diseases	laurence_holding@moh.govt.nz
**Nicaragua**	Arelisabel Ruiz Guido	Coordinadora del<br>Programa Nacional de Tuberculosis	coordtuberculosis@minsa.gob.ni
**Niger**	Dr Assao Neino Mourtala Mohamed	Coordonnateur,<br>National Programme tuberculose	mourt2000@yahoo.fr
**Nigeria**	Adebola Lawanson	National Coordinator NTBLCP	oluwadarasimi22@gmail.com
**North Macedonia**	Biljana Ilievska Poposka	NTP maneger	biljana.ilievska@yahoo.com
**Northern Mariana Islands**	Ngoc-Phuong Luu	CHCC Medical Director of Public Health	phuong.luu@dph.gov.mp
**Oman**	Fatma Alyaquobi	Dr	fatmayaquobi@yahoo.com
**Pakistan**	Aurangzaib Quadir Baloch	Deputy National Coordinator - TB	draurangzaib@ntp.gov.pk
**Palau**	Connie Olikong	TB Program Manager	connie.olikong@palauhealth.org
**Panama**	Edwin Aizpurúa	Jefe del Programa Nacional<br>de controlde la Tuberculosis	dr.edwinaizpuruajr@gmail.com
**Papua New Guinea**	Paul Aia	National TB Program Manager	koltas44@gmail.com
**Paraguay**	Sarita Aguirre	Directora	sarita.aguirre79@yahoo.com
**Peru**	JULIA ROSA MARIA RIOS VIDAL	DIRECTORA EJECUTIVA	jriosv@minsa.gob.pe
**Philippines**	Anna Marie Celina Garfin	Program Manager	garfinamc@gmail.com
**Poland**	Stefan Wesołowski	Director Nat. TB and Lung Dis. Res.Inst.	instytut@igich.edu.pl
**Portugal**	Isabel Carvalho	MD	carvalho.isabel@gmail.com
**Puerto Rico**	Olga Joglar	Program Director	olga.joglar@salud.pr.gov
**Qatar**	Abdullatif Al Khal	NTP manager	aalkhal@hamad.qa
**Republic of Korea**	Kong Insik	Director,Division of TB and HIV/AIDS Control	insik.kong@korea.kr
**Republic of Moldova**	Valentina Vilc	Deputy director, Institute of<br>Phthisiopneumology "Chiril Draganiuc"	valentina_vilc@yahoo.co.uk

**Romania**	Ioana Munteanu	Natioanal Coordinator of TB Prevention, Sureveillance and Control Program	ioana.munteanu2015@yahoo.ro
**Russian Federation**	Irina Vasilyeva	Chief TB expert of the Russian Ministry of Health	vasil39@list.ru
**Rwanda**	Dr. Patrick MIGAMBI	Manager, Tuberculosis and Other Communicable Respiratory Diseases Division	patrick.migambi@rbc.gov.rw
**Saint Kitts and Nevis**	Dwain Archibald	Focal Point of Tuberculosis in St.kitts	dwain.archibald.doc@gmail.com
**Saint Lucia**	Gail Gajadhar	National TB Programme Manager	gajadharg@hotmail.com
**Saint Vincent and the Grenadines**	Ferosa Roache	Director, HIV/AIDS/STI Prevention and Control	ferosaroache@hotmail.com
**Samoa**	Serafi Moa	Senior Nurse, Communicable Disease Clinic	serafim@nhs.gov.ws
**San Marino**	Gabriele Rinaldi	Health Authority Director	gabriele.rinaldi@pa.sm
**Sao Tome and Principe**	Bonifacio SOUSA	Coordonateur PNLT	bonifaciosousa1@yahoo.com.br
**Saudi Arabia**	ahmed hakawy	General Director of Communicable Diseases prevention and control	ahakawi@moh.gov.sa
**Senegal**	Dr Marie Sarr Diouf	Coordonnateur du programme national de lutte contre la tuberculose	drmariesarr@yahoo.fr
**Serbia**	Violeta Mihailovic Vucinic	Full Proffesor of Internal Mediicine, School of Medicine, Belgrade University	violetavucinic@gmail.com
**Seychelles**	Louine Morel	SEnior Medical Registrar	louine.morel@health,gov.sc
**Sierra Leone**	Dr Lynda Foray	Program Manager	lynforar@gmail.com
**Singapore**	Cynthia Chee	Director, Singapore TB Elimination Programme (STEP)	Cynthia_Chee@ttsh.com.sg
**Sint Maarten (Dutch part)**	Henry Maria	Section Head, General Health Care of Collective Prevention Services	maria.henry@sintmaartengov.org or sectionpep@yahoo.com
**Solomon Islands**	Noel Itogo	National Coordinator	NItogo@moh.gov.sb
**Somalia**	Sindani Ireneaus Sebit	Medical Officer	sindanii@who.int
**South Africa**	Lerole David Mametja	TB Cluster Manager	david.mametja@health.gov.za
**South Sudan**	Martin Likambo	Acting TB Programme Manager	martinlikambo@gmail.com
**Sri Lanka**	Nirupa Pallewatte	Director (Acting)	ddnptccd@health.gov.lk

**Sudan**	Mousab Elhag	Director of Communicable and Non-Communicable Diseases Control Directorate	mousabsiddig@gmail.com
**Suriname**	Eric Commiesie	coordinator TB program	ercom75@gmail.com
**Switzerland**	Aylin Jaspersen	Medical Officer	aylin.jaspersen@bag.admin.ch
**Syrian Arab Republic**	HAZAR ZUHEIR FAROUN	Director Of Communicable and non Communicable Diseases Department	hazarfaroun@yahoo.com
**Tajikistan**	Аслиддин Раджабзода	Директор	asliddin.81@mail.ru
**Thailand**	Phalin Kamolwat	Director, Bureau of Tuberculosis	phalin1@hotmail.com
**Timor-Leste**	Constantino Lopes	National Program Manager	costa_tb@yahoo.com
**Togo**	Anoumou Yaotsè Prof DAGNRA	Coordonnateur par intérim	claverdagnra@gmail.com
**Tonga**	Dr Louise Fonua	TB Programme Manager	lsfonua@gmail.com
**Trinidad and Tobago**	Dr Michelle Trotman	Thoracic Medical Director (Ag)	tmd.caura@ncrha.co.tt
**Tunisia**	DHIKRAYET GAMARA	NTP MANAGER	dhikrayet.gamara@rns.tn
**Turkey**	ERHAN KABASAKAL	MD, Head of Tuberculosis Department	erhankabasakal@gmail.com
**Turkmenistan**	Айсолтан Чарыева	Заместитель Генерального Директора Центра инфекционных болезней по центру профилактики и лечения туберкулеза	tub.merk@mail.ru
**Turks and Caicos Islands**	Jackurlyn Sutton	Chief Nursing Officer Primary Health Care Manager	jsutton@gov.tc
**Tuvalu**	Christine Lifuka	National TB Coordinator	dinedemanzanillo@gmail.com
**Uganda**	Turyahabwe Stavia	Ag. Assistant Commissioner TB and Leprosy Control	turyahabwestavia@gmail.com
**Ukraine**	Яна Терлеева	Начальник отдела координации программ лечения ТБ	i.terleieva@phc.org.ua
**United Arab Emirates**	NADA ALMARZOUQI	DIRECTOR, PREVENTIVE MEDICINE DEPARTMENT	nada.almarzouqi@mohap.gov.ae
**United Republicof Tanzania**	Beatrice Mutayoba	Programme Manager	beatricemutayoba@yahoo.com
**United States**	Adam Langer	Surveillance Team Lead	akl7@cdc.gov
**Uruguay**	Arrieta Pessolano Dr. Fernando	Director Dpto. Tuberculosis CHLA-EP	farrieta@chlaep.org.uy drfernando.arrieta@gmail.com
**Uzbekistan**	Наргиза Парпиева	Директор РСНПМЦФиП	nargiza.parpieva@minzdrav.uz

**Vanuatu**	Edna Iavro	Acting National TB Program Manager	eiavro@vanuatu.gov.vu
**Venezuela (Bolivarian Republic of)**	Mercedes España Cedeño	Coordinador de la División de Salud Respiratoria	programatbvenezuela@yahoo.com
**Viet Nam**	VIET NHUNG NGUYEN	Manager, National TB control programme	vietnhung@yahoo.com
**Wallis and Futuna Islands**	Patrick LAMBRUSCINI	DIRECTEUR AGENCE DE SANTE WALLIS ET FUTUNA	patrick.lambruscini@adswf.fr
**West Bank and Gaza Strip**	Diaa Hjaija	director of communicable disease department	pmdmoh@yahoo.com
**Yemen**	Dr. Esam Moammed Mahyuob Alsabery	Manager of National TB control program	esam_mahyoub@yahoo.com
**Zambia**	DR LUNGU PATRICK SAILI	NTP MANAGER	Patrick Lungu patrickpj456@yahoo.co.uk
**Zimbabwe**	Charles Sandy	Deputy Director (TB Control)	sandycrm@yahoo.com
